# Preservation of One Anterior and One Posterior Internal Iliac Artery Branch in a Case of Bilateral Common and Internal Iliac Arterial Aneurysms

**DOI:** 10.1016/j.ejvsvf.2025.05.009

**Published:** 2025-05-24

**Authors:** Anna Stene Hurtsén, Artai Pirouzram, Tal Hörer

**Affiliations:** a Department of Cardiothoracic and Vascular Surgery, Faculty of Medicine and Health, Örebro University, Örebro, Sweden; b Department of Cardiothoracic and Vascular Surgery, Linköping University Hospital, Linköping, Sweden

**Keywords:** Internal iliac aneurysm, Common iliac aneurysm, Iliac branch device, Pelvic circulation

## Abstract

**Introduction:**

Endovascular treatment of iliac aneurysms with sparing of the internal iliac arterial circulation is feasible with iliac branch devices. However, insufficient distal seal with the endovascular devices on the market can be challenging. In this case, the anatomy was complex due to the extent of the aneurysms, and the available technical options were limited.

**Report:**

A 65 year old man with aneurysms in the left common iliac (43 mm) and bilateral internal iliac arteries (right 41 mm; left 49 mm) was treated with an aortobi-iliac stent graft and bilateral iliac branch devices with extensions to opposing anterior (right) and posterior (left) branches of the internal iliac artery through staged interventions. At six weeks of follow up all treated aneurysms had decreased or were stable in size. Clinical signs of right sided gluteal claudication were evident at six weeks of follow up but no symptoms remained 20 weeks post-operatively.

**Discussion:**

The presented case illustrates a technique to preserve pelvic circulation in a case of bilateral common and internal iliac arterial aneurysms. Extensions of the internal iliac limb of the iliac branch device, into the opposing anterior and posterior divisions of the internal iliac artery, may offer a strategy to reduce pelvic ischaemia in scenarios where the anatomy limits the use of standard iliac branch devices.

## Introduction

Isolated common iliac arterial aneurysms (CIAAs) are relatively rare (7% of intra-abdominal aneurysms) and the internal iliac artery (IIA) is involved in 10–30% of cases. Up to 50% of CIAAs are bilateral.^1^ The primary objective of treatment is to exclude the aneurysm sac while ensuring perfusion to at least one IIA to avoid pelvic ischaemia. A problem with bilateral disease is when the IIA extends to the bifurcation of branches on both sides, which makes preservation of both branches difficult. This report details a staged endovascular management of bilateral CIAAs and internal iliac arterial aneurysms (IIAAs) using a modified approach involving bilateral iliac branch devices (IBD), with internal iliac extensions directed into opposing anterior and posterior divisions of the IIA to maintain pelvic circulation in as favourable a way as possible.

## Report

**Figure 1:**
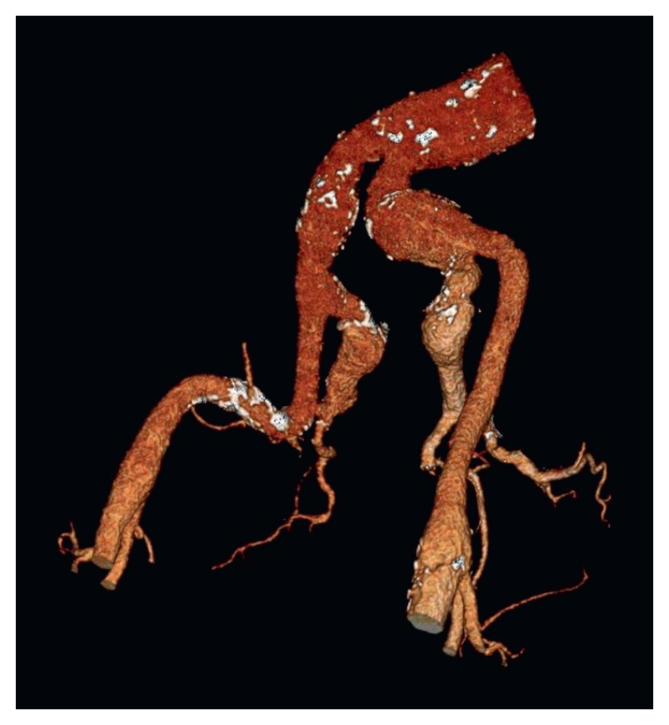
Anatomy of the iliac arteries.

In a 65 year old man recently diagnosed with left ventricular heart failure, bilateral CIAAs and IIAAs (right CIA diameter 27 mm; left CIA diameter 43 mm; right IIA diameter 41 mm; left IIA diameter 49 mm) and an infrarenal abdominal aortic aneurysm (44 mm) were found on a computed tomography (CT) examination incidentally (Fig. 1). The patient was physically active and had no symptoms from cardiac failure or of claudication. Given the size of the IIAAs an intervention was indicated subacutely. The CT showed a complex pelvic anatomy with IIAAs extending into the bifurcation of the anterior and posterior branches with no landing zone in the IIA on either side. A decision to spare the posterior branches on one side and the anterior branches on the other side was made, in a two staged intervention, with the completion of an endovascular aneurysm repair (EVAR) in the last instance.

**Figure 2:**
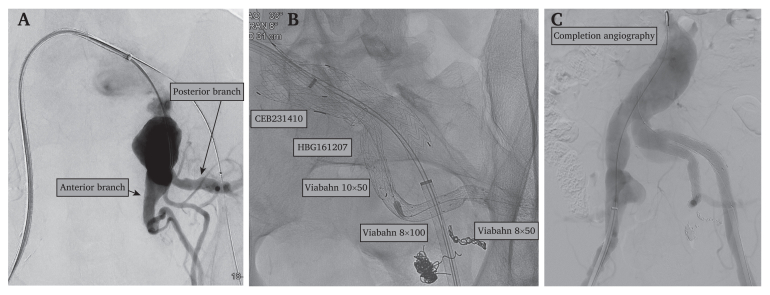
Selective angiography of the left iliac artery (A), endoprostheses used (B) and completion angiography (C).

In the first stage, preservation of the posterior branch of left IIA was intended. Coiling of the anterior branches of the left IIA using Ruby coils (standard and packing types; Penumbra, Alameda, CA, USA) was performed. In the posterior branch an 8 × 50 mm Viabahn (Gore Medical, Newark, DE, USA) stent graft was deployed (Fig. 2). Subsequently, a third branch was coiled at the posterior branch's origin using Ruby coils. An excluder endoprosthesis iliac branch endograft (IBE) (CEB231410, Gore) was deployed into the left CIA (Fig. 2). Additionally, distally to proximally from the posterior branch, an 8 × 100 mm Viabahn stent graft (Gore Medical) and an intermediate Viabahn (Gore Medical) 10 × 50 mm followed by an Excluder endoprosthesis (HBG161207, Gore Medical) were deployed and connected to the IBE (Fig. 2). An Excluder (PLC161000, Gore) abdominal aortic aneurysm (AAA) endoprosthesis stent graft was deployed in the external iliac artery (Fig. 2). The IBD graft had migrated on completion angiography and did not seal the CIA (Fig. 2). However, the IIAA appeared excluded. The patient was discharged on post-operative day one on acetylsalicylic acid (Trombyl) 75 mg a day.

**Figure 3:**
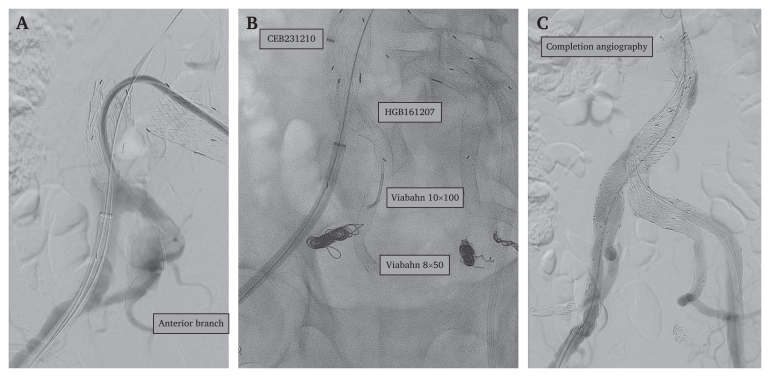
Selective angiography of the right iliac artery (A), endoprostheses used (B) and completion angiography (C).

In stage 2 the anterior branches of the right IIA were preserved. The posterior branches of the right IIA were occluded with a POD 8 coil (Penumbra) and standard coils (Penumbra). An IBE (CEB231210, Gore) was deployed into the right CIA (Fig. 3). An 8 × 50 mm Viabahn (Gore Medical) stent graft (Fig. 3) was deployed distally in the anterior branch, followed proximally by a 10 × 100 mm Viabahn (Gore Medical), and finally an Excluder endoprosthesis (HBG161207, Gore Medical) IBD graft (Fig. 3). The procedure then proceeded with an EVAR (Excluder AAA endoprosthesis RLT281412, Gore Medical) below the left renal artery, follow by leg endoprosthesis (PLC161200 and PLC271000, Gore Medical) (Fig. 3). From the contralateral left iliac branch an additional 27 mm Excluder AAA endoprosthesis (PLC271000, Gore Medical) was deployed as a bridge between the IBD and EVAR graft. Completion angiography showed a minor type 1A endoleak. Ballooning was repeated, but no further extension was made due to the long proximal neck.

The patient was discharged on the second post-operative day without complications. Six weeks post-operatively, a type 2 endoleak in the right IIA (no expansion of the aneurysm sac) and clinical signs of right sided buttock claudication were present. At 20 weeks post-operatively no symptoms of buttock claudication or erectile dysfunction remained and all aneurysm sacs were diminished. No endoleak was observed.

## Discussion

The described case demonstrates a technique to preserve pelvic circulation when the anatomy makes standard IBD hard to plan. Challenges with insufficient distal seal remain in certain cases when treating iliac aneurysms with the endovascular devices available on the market. In this case the anatomy was challenging with the extent of aneurysms, and the technical options were limited.

When IIAs are plugged bilaterally, the risk of pelvic, colonic, and spinal ischaemia is not negligible.^2^, ^3^, ^4^, ^5^ In a systematic review and meta-analysis of IIA exclusion for patients undergoing EVAR, buttock claudication occurred in one in four cases.^3^ For unilateral IIA occlusion, the risk of buttock claudication is approximately 15%, and in patients undergoing bilateral IIA occlusion the risk is estimated to be 23%.^2^ Erectile dysfunction is present in about 10.2% of male cases.^3^ Iliac branch devices are nowadays commonly used in the treatment of aorto-iliac and isolated IIAAs with a high success rate, with a primary patency of the IIA limb estimated to be 95%.^6^ The risk of buttock claudication post-operatively can be reduced by preservation of the IIAs if anatomically and technically feasible.^7^ Although IBDs have enhanced the feasibility of IIA preservation, their use is restricted to a specific range of aneurysm anatomies. In the present case, bilateral occlusion of the IIAs would be unfavourable with respect to the relatively young age of the patient. The IIAs extended into both main branches of the IIA, and preserving both branches was considered impossible with an acceptable expected patency. When planning the case, the available published data were reviewed, but limited information was found. The landing zone (diameter, continuity, length, configuration, etc.) parameters are important when selecting the target vessel for landing. The aim was to treat both sides, and anatomically and technically, all combinations were feasible and achievable. Considering the patient's age and factors like sexual function, it was decided to extend the internal iliac component to one anterior and one posterior branch. However, different combinations could have been performed.

A staged approach was selected mainly due to extensive disease with unpredictable procedure duration. At least one prior case with preservation of pelvic circulation by extending opposing posterior and anterior divisions of the internal iliac branch has been described.^8^ In this case, all stent grafts were patent and all aneurysms had decreased or were stable in size at the one month follow up. Furthermore, the patient was asymptomatic and no clinical signs of pelvic ischaemia were present at the six month follow up. In the present case, right sided gluteal claudication was evident at the first post-operative visit at six weeks. The main difference between these cases is the age of the patient, where the patient was 18 years younger in the present study, probably enjoying a more active lifestyle. However, buttock claudication may resolve over time (48% after 21.8 months)^3^ and was not present at the later follow up in the present case. In another case of bilateral CIAAs and IIAAs both posterior branches of the IIA were preserved, and no signs of buttock claudication was observed.^9^ However, erectile function was not stated in that case.

The long term patency of the internal iliac limb using the described technique remains uncertain and necessitates further investigation with extended follow up studies. In conclusion, extensions of the internal iliac limb of the IBD into opposing anterior and posterior divisions of the IIA may provide an approach for reducing pelvic ischaemia when managing concurrent CIAAs and IIAAs.

## References

[1] Wanhainen A., Verzini F., Van Herzeele I., Allaire E., Bown M., Cohnert T. (2019). Editor's Choice – European Society for Vascular Surgery (ESVS) 2019 clinical practice guidelines on the management of abdominal aorto-iliac artery aneurysms. Eur J Vasc Endovasc Surg.

[2] Fujioka S., Hosaka S., Morimura H., Chen K., Wang Z.C., Toguchi K. (2017). Outcomes of extended endovascular aortic repair for aorto-iliac aneurysm with internal iliac artery occlusion. Ann Vasc Dis.

[3] Bosanquet D.C., Wilcox C., Whitehurst L., Cox A., Williams I.M., Twine C.P. (2017). Systematic review and meta-analysis of the effect of internal iliac artery exclusion for patients undergoing EVAR. Eur J Vasc Endovasc Surg.

[4] Warein E., Feugier P., Chaufour X., Molin V., Malikov S., Bartoli M.A. (2016). Amplatzer plug to occlude the internal iliac artery during endovascular aortic aneurysm repair: a large multicenter study. Eur J Vasc Endovasc Surg.

[5] Kouvelos G.N.K.A., Antoniou G.A., Oikonomou K., Verhoeven E.L. (2016). Outcome after interruption or preservation of internal iliac artery flow during endovascular repair of abdominal aorto-iliac aneurysms. Eur J Vasc Endovasc Surg.

[6] Schneider D.B., Matsumura J.S., Lee J.T., Peterson B.G., Chaer R.A., Oderich G.S. (2023). Five-year outcomes from a prospective, multicenter study of endovascular repair of iliac artery aneurysms using an iliac branch device. J Vasc Surg.

[7] Cao Z., Zhu R., Ghaffarian A., Wu W., Weng C., Chen X. (2022). A systematic review and meta-analysis of the clinical effectiveness and safety of unilateral versus bilateral iliac branch devices for aortoiliac and iliac artery aneurysms. J Vasc Surg.

[8] Al-Hakim R., Watch L., Powell A. (2018). Endovascular treatment of concurrent bilateral common and internal iliac artery aneurysms with preserved pelvic circulation: bilateral iliac branch devices with opposing single division internal iliac artery sparing. J Vasc Interv Radiol.

[9] Tajima Y., Goto H., Akamatsu D., Serizawa F., Suzuki S., Horii S. (2022). Prevention of buttock claudication by preserving antegrade bilateral superior gluteal arterial blood flow in EVAR for aorto-iliac aneurysm accompanied by bilateral internal iliac artery aneurysms. Ann Vasc Dis.

